# Artificial Kidney Engineering: The Development of Dialysis Membranes for Blood Purification

**DOI:** 10.3390/membranes12020177

**Published:** 2022-02-02

**Authors:** Yu-Shuo Tang, Yu-Cheng Tsai, Tzen-Wen Chen, Szu-Yuan Li

**Affiliations:** 1 Department of Internal Medicine, Wan Fang Hospital, Taipei Medical University, Taipei 116, Taiwan; 110136@w.tmu.edu.tw; 2 Department of Medical Research, Taipei Veterans General Hospital, Taipei 112, Taiwan; tsai829@hotmail.com; 3 Division of Nephrology, Department of Medicine, Wei-Gong Memorial Hospital, Miaoli County 351, Taiwan; twchen@tmu.edu.tw; 4 Division of Nephrology, Department of Medicine, Taipei Veterans General Hospital, Taipei 112, Taiwan; 5 School of Medicine, National Yang-Ming University, Taipei 112, Taiwan

**Keywords:** artificial kidney, nanopore silicon membrane, REDY system, cell bioreactor, wearable artificial kidney, implantable artificial kidney

## Abstract

The artificial kidney, one of the greatest medical inventions in the 20th century, has saved innumerable lives with end stage renal disease. Designs of artificial kidney evolved dramatically in decades of development. A hollow-fibered membrane with well controlled blood and dialysate flow became the major design of the modern artificial kidney. Although they have been well established to prolong patients’ lives, the modern blood purification system is still imperfect. Patient’s quality of life, complications, and lack of metabolic functions are shortcomings of current blood purification treatment. The direction of future artificial kidneys is toward miniaturization, better biocompatibility, and providing metabolic functions. Studies and trials of silicon nanopore membranes, tissue engineering for renal cell bioreactors, and dialysate regeneration are all under development to overcome the shortcomings of current artificial kidneys. With all these advancements, wearable or implantable artificial kidneys will be achievable.

## 1. History of the Artificial Kidney

The artificial kidney, also known as a dialyzer, is an unreplaceable part of current renal replacement therapy. Without dialysis and kidney transplantation, end stage renal failure meant the termination of a patient’s life in the past. The technology of dialysis has been developed for decades since the early 20th century. In 1928, Haas used a cellulose membrane to treat six patients with dialysis with the anticoagulant of hirudin [1,2]. It ended with no patient survival because of the immature technique.

In 1943, Dutch physician Willem Kolff invented the first practical dialyzer [3], the rotating drum kidney, which was composed of a 20-m-long rotating cellophane tube wrapped around a 2 m horizontal drum as the semipermeable membrane. A 67-year-old patient with acute kidney failure in the course of an exacerbated bile duct infection was treated with the rotating drum kidney successfully without subsequent renal impairment. This device could remove uremic toxins but not excessive body fluid, mainly because the transmembrane pressure produced by gravitation is not sufficient to provide ultrafiltration. Thus, renal failure with fluid-overload, such as pulmonary edema or hypertension, was still untreatable with the rotating drum kidney. 

After the first successful use of dialysis, the artificial kidney developed rapidly. A modified dialyzer withstanding higher transmembrane pressure allowed excessive fluid removal [4], a parallel plate dialyzer [5] for lower blood flow resistance and more controllable ultrafiltration, subtractions of heparin [6] as anticoagulant for dialysis, and advanced shunt for steady blood vessel assessment [7] all contributed significantly to the development of renal replacement therapy. In 1960, the first chronic dialysis facility was established in Seattle, and end stage renal disease became no longer a fatal disease.

Nowadays, hemodialysis is a standard treatment all over the world for millions of patients with various diseases from acute kidney injury to end stage renal disease. The hollow-fibered dialyzer and more efficient machines replaced the old giant rotating drum kidney. The healthcare industry keeps making new dialyzers to improve patients’ lives.

## 2. Designs of the Modern Artificial Kidney

Although the new blood purification system is much better in terms of performance, efficiency, and biocompatibility, the basic idea of blood purification is not different from the first-generation rotating drum kidney. The primary goals of dialyzers are removing solutes and excessive fluid from the blood of the patients. To achieve these goals, the designs of dialyzers are based on four fundamental aspects: structure, performance, biocompatibility, and membrane material.

### 2.1. Structure

**Figure 1:**
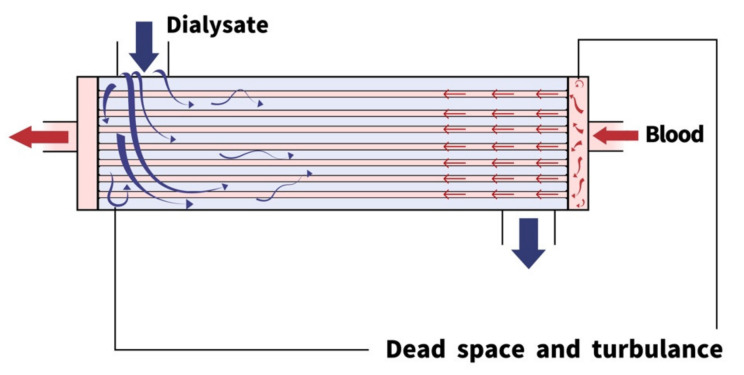
Modern design of the artificial kidney: the geometric designs of the modern artificial kidney are predominantly hollow-fibered. Low fluid resistance and expanded surface area facilitated the efficiency of dialysis. However, the complicated structure creates multiple dead spaces and turbulence within both compartments.

Modern artificial kidneys are hollow-fibered dialyzers composed of a blood compartment, dialysate compartment, and semipermeable hollow fibers (Figure 1). Structures of each part of dialyzers influence the fluid mechanism significantly [8]. Theoretically, a high clearance could be achieved by reduced fluid resistance, membrane thickness, and expanded surface area.

The geometric designs have evolved greatly since the first rotating drum kidney, which was a 20-m-long cellophane tube wrapped around a 2 m horizontal drum and soaked in a tank of dialysate. In contrast, modern dialyzers are hollow-fibered and much smaller. The hollow-fibered structures provide better membrane surface area and less fluid resistance [8,9]. The hollow-fibered design also makes it easier to control pressure in the blood compartment to achieve precise ultrafiltration [9,10].

There are, however, several drawbacks of hollow-fibered dialyzers. The hollow-fibered structure creates complicated space in the dialysate compartment, which produces turbulence and dead space [8] and impairs the dialysis efficiency and decreases the predictability of each dialyzer’s performance. In clinical practice, the hollow-fibered structure requires careful deaeration before dialysis treatment to prevent air bubbles from staying in the blood compartment [11]. Considering the advantages of hollow-fibered dialyzers, modern artificial kidneys are still mostly hollow-fibered.

### 2.2. Performance

The performance of each dialyzer depends on its ability to remove solutes and excessive fluid from the patient’s blood. In regard to blood purification, solutes in the plasma could be classified based on their molecular weights [12]. In healthy human bodies, small molecular weight solutes (less than 500 Daltons), such as glucose, electrolytes, lipids, urea, and creatinine, are removed by the kidney through ion channels or diffuse across the cell membrane directly. Middle molecular weight solutes (500–15 k Daltons), such as hemoglobulin, β2-microglobulin, and bilirubin, metabolize in human bodies through various pathways. Large molecular weight solutes (greater than 15 k Daltons) cannot pass through most membranes, which create oncotic pressure across semipermeable cell membranes. 

To replace the function of the kidney, dialyzers are designed specifically for solutes with different molecular weights. The semipermeable membrane creates the boundary between blood and dialysate. Solutes with different molecular weights behave differently across the membrane on the basis of three main types of clearance: diffusion, convection, and adsorption.

#### 2.2.1. Diffusion

Diffusion is the random movement of particles from a region of high concentration to a region with low concentration. Dialyzer efficiency, the permeability of small molecular weight solutes, is determined by membrane surface area, physical characteristics of the membrane, and temperature [13]. Since the efficiency remains the same for a specific artificial kidney under fixed temperature. Diffusion is mainly determined by the concentration gradient across the membrane, which is influenced by blood and dialysate flow rates. Diffusion predominantly affects small molecular weight solutes such as electrolytes and creatinine. Clearance of middle molecular weight solutes via diffusion is usually unachievable.

#### 2.2.2. Convection

Convection describes the movement of particles within fluid. The convective clearance or beta-2-microglobulin clearance of a dialyzer, commonly referred to as membrane flux, represents the water flux or permeability of middle molecular weight molecules. In an artificial kidney, the major determinants are ultrafiltration triggered by transmembrane pressure [14].

Ultrafiltration is a milestone for the development of dialysis, which allows the removal of excessive fluid in uremic patients. Moreover, solutes are also transported through the membrane during ultrafiltration. This phenomenon is called solvent drag. Most middle molecular weight solutes are removed through this mechanism.

#### 2.2.3. Adsorption

Adsorption occurs with the deposition of proteins on the membrane during dialysis, which creates a biofilm over the inner surface of hollow fibers. The involved particles are mostly ranged from middle to large molecular weight solutes. Adsorption clearance would be affected by membrane porosity and charge-dependent selective permeability [15].

### 2.3. Biocompatibility and Systemic Effects

During dialysis, interactions between blood and the inner surface of hollow fibers might trigger systemic effects including inflammation [16,17], coagulation [18,19], erythropoietin resistance [20], and protein malnutrition [21].

Adsorption during dialysis creates a biofilm which triggers coagulation and inflammation reactions. Coagulation, induced by adsorbed fibrinogen, increases the risk of intra-dialyzer clotting and post-dialysis thrombocytopenia [19]. Inflammation, triggered by adsorbed C3b and the activated complement pathway [16], would induce cytokine release, create oxidative stress, and result in epithelial cell injury, arthrosclerosis, and transient granulocytopenia after dialysis [22,23]. Erythropoietin resistance and protein malnutrition are also induced by similar interactions.

In addition to coagulation and inflammation, other systemic effects such as intra-dialysis hypotension, hemodialysis induced amyloidosis, and residual renal function loss are all undesirable complications of blood purification treatment. Improved biocompatibility is one of the most important goals in future dialyzer development.

### 2.4. Membrane Material and Characteristics 

As an essential part of the artificial kidney, the hollow fiber membrane divides blood and dialysate compartments with different filtration characteristics. The membrane materials used in artificial kidneys include cellulose derivatives, polysulfone derivatives (PSU), polyacrylonitrile (PAN), polymethylmethacrylate (PMMA), and ethyl vinyl-acetate copolymer (EVAL) [24]. The initial hollow-fibered membranes were mostly built with cellulose derivatives, but synthetic polymer gradually became the major material because of better biocompatibility and performance. The hydrophilicity of membranes is a major determinant of biocompatibility. Hydrophobic synthetic polymer membranes increase the risk of platelet adhesion [18], Nevertheless, various addictive materials have been doped in the synthetic materials to improve the biocompatibility [25]. 

The semipermeable properties of membranes are mostly determined by pore size, porosity distribution, and thickness, which can be investigated with various methods, such as electron microscopy, gas adsorption, X-ray diffraction, etc. [26,27]. At present, various membranes for biomedical engineering such as artificial lungs, plasma exchange therapy, hemofiltration membranes, and hemoadsorption membranes are contributing to medical treatment around the world. Extracorporeal membrane oxygenation (ECMO) and the artificial liver or pancreas are all possible applications of membranes [28]. 

## 3. Room for Improvement

The development of artificial kidneys and semipermeable membranes for blood purification is still ongoing. The advanced synthetic technique, nanotechnology, accumulated data, and experience of dialysis all contributed to the potential of future dialyzer development.

The current system of blood purification became mature after decades of development. From regular dialysis for end-stage renal disease to continuous venovenous hemofiltration (CVVH) for acute kidney injury in intensive care units, various indications are widely used clinically all over the world. However, there is still plenty of room for improvement. There are three main aspects of problems for the current artificial kidneys: patient’s quality of life, complications of artificial kidneys, and lack of metabolic functions.

### 3.1. Patient’s Quality of Life

The restriction of life is one of the most unbearable side effects of long-term dialysis. Renal replacement therapy requires three 4-h-long dialysis courses every week. The time-consuming course, frequent hospital visiting, water and food restrictions all reduce the patient’s quality of life, joy of tasting, and freedom of scheduling [29].

To improve patient’s quality of life, the system of dialysis must be modified greatly. Home dialysis, wearable devices, and even implantable artificial kidneys are all possible solutions. To achieve this goal, the source of dialysate, fresh water supply, energy supplement, the size of the artificial kidney, and financial costs are all challenges on the path toward artificial kidneys that provide better patient quality of life.

### 3.2. Complications of Artificial Kidneys

The problems of biocompatibility and systemic effects are usually accompanied with hemodialysis. Multiple dialysis-associated complications, such as intra-dialysis hypotension, dialysis-related amyloidosis, and erythropoietin resistance, reflect the fact that the current designs of artificial kidneys require further modification and membrane engineering [30].

The material compositions of hollow-fibered membranes evolved from the original cellophane to modern synthetic polymer materials or cellulose. Water affinity, a significant determinant of membrane biocompatibility, is one important difference between cellophane and synthetic materials. Hydrophilic materials, such as cellophane, induces the complement pathway more easily. Whereas intra-dialysis coagulation is more common with the hydrophobic inner surface of hollow-fibered membranes [18]. Hollow-fibered membranes with better biocompatibility could be created by modifying hydrophilicity with modern synthetic technology.

Another method of improving biocompatibility is by adjusting the inner membrane surface with coating materials, such as heparin or vitamin E. Theoretically, heparin can reduce coagulation reaction and vitamin E is able to improve oxidative stress induced by inflammation. Parameters of oxidative stress, erythropoietin resistance, and nutritional status were improved with vitamin E coated membranes in some studies [31,32,33]. However, the significance of those coating materials remains debatable [34,35].

Other modifications, such as membrane porosity and geometric designs, can all influence the biocompatibility of hollow-fibered membranes. Although being reduced, complications of artificial kidney still limit the capability of hemodialysis after decades of development.

Rapid fluid removal three times a week, hemodynamic change, oxidative stress, and blood vessel calcification all contribute to the high cardiovascular risk in patients with end stage renal disease. Prolonged dialysis duration with nocturnal hemodialysis has been proved to improve cardiac hypertrophy, blood pressure, and heart function [36,37]. However, nocturnal hemodialysis is practical only if home dialysis or wearable artificial kidneys are easily available for every suitable patient.

### 3.3. Lack of Metabolic Functions

Natural kidneys are not just organs of filtration, they are also responsible for several metabolic functions, such as synthesis of 1,25-dihydroxyvitamin D (1,25(OH)2 D) [38], ammoniagenesis [39,40], glutathione metabolism [41], erythropoietin production [42], and immunoregulatory support [43]. Modern designs of artificial kidneys put much emphasis on the efficiency of dialysis and fluid removal but not these metabolic functions. To fully replace renal function, the metabolic functions of the kidney must not be neglected in future developments.

## 4. Direction of Future Artificial Kidney Development

Although the system of hemodialysis with hollow-fibered membranes is well established, the designs of artificial kidneys are still evolving. There are two major trends of future development: miniaturization and restoration of metabolic functions.

Miniaturization of artificial kidneys is one of the major goals of recent dialyzer development, which enables much easier manipulation of dialysis and allows further wearable or implantable designs of artificial kidneys. To achieve miniaturization, membrane engineering for more efficient membranes is essential. Many projects are currently under development [44,45,46,47,48,49,50,51,52,53]. With the convenience of wearable or implantable artificial kidneys, patients can have frequent and prolonged dialysis to reduce hemodynamic impact during dialysis courses, which have been proven to improve survival, hospitalization rates, bone mineral metabolism, and medication burden [36,37,54,55,56].

Metabolic functions of natural renal tubular cells are not provided by current artificial kidneys, such as biosynthesis of 1,25-dihydroxyvitamin D (1,25(OH)2 D) [38], ammoniagenesis [39], glutathione metabolism [57], and immunoregulatory support [43]. Erythropoietin production is provided by renal interstitial cells. Without those metabolic functions, abnormal calcium metabolism, ectopic calcification, and oxidative stress significantly increase morbidity and mortality in end-stage renal disease patients [43].

## 5. Key Considerations for Future Dialyzer Membrane Development

Multiple projects and innovative designs for artificial kidneys and membranes are still evolving. To reach the goals of miniaturization and restoration of renal metabolic functions, several technologies could be the key for future dialyzers.

### 5.1. Nanotechnology

Nanotechnology is widely used in the fields of medicine, consumer products, energy, materials, and manufacturing. However, the application of nanotechnology to blood purification treatment was relatively late compared to other industries.

Microelectromechanical systems (MEMS) are a form of fabrication technology that originated from the microelectronic industry [58]. Silicon, polymers, or metals could all be fabricated precisely to the scale of nanometers by deposition, lithography, etching process, and sputtering. Conventional artificial dialyzers mostly consist of semipermeable hollow fibers with wide pore size distribution. The pore size variation limits the selectivity and permeability of conventional semipermeable membranes. With microelectromechanical systems, semipermeable membranes with identical pore size could be manufactured. Microelectromechanical systems also allow chemistry modification of membranes to adjust electrostatic charge and further prevent platelet adhesion or protein adsorption [59,60,61,62], which improves the biocompatibility of membranes significantly. The silicon nanopore membranes modified with polyethylene glycol (PEG) and polyvinylamine (PVAm) polymers showed excellent hemocompatibility in the aspects of surface coagulation, complement and platelet activation, and adhesion [63], which makes silicon nanopore membranes an option for implant applications.

Silicon nanopore membranes for artificial kidneys have been developed for decades [64,65,66]. Scientists have developed a silicon nanopore membrane with an array of rectangular pore slits measuring 2.3 µm × 11 nm with an effective diffusion length of 100 µm [64]. The rectangular pore was designed to mimic the slit between natural kidney podocytes, which enables higher hydraulic permeability compared with conventional hollow-fibered membranes [66,67,68]. Compared with current dialyzers, the silicon nanopore membrane provides a comparable urea clearance under 1/20th of the blood flow rate [64]. The extremely low requirement of blood flow allows blood purification without a mechanical pump as the patient’s nature circulation is sufficient to drive the system. With an 8 h daily dialysis course and the target of a standard Kt/V(K: urea clearance, t: time of dialysis, V: volume distribution of urea) of 2.0 per week, only 0.17 m^2^ of silicon nanopore membrane surface area is required, which is 10 times less than current dialysis membranes [64]. Uniform pore size also provides strict molecular weight cut-off values [64] and better middle molecular weight solutes clearance. The nanopore membrane has a 18% beta-2-microglobulin clearance rate, which is much higher than current high-flux dialyzers [64].

With the aid of nanotechnology, membranes with uniform pore size and extremely thin silicon structures provide better clearance under extremely low blood flow rates. Biocompatibility and clearance of middle molecular weight solutes are also improved. With these improvements, the development of wearable or implantable artificial kidneys is possible.

### 5.2. Tissue Engineering

Tissue engineering combines the use of cells and materials to create artificial structures with specific bioactivity [69]. Selective cells are attached to designed scaffolds and further implanted to animal or human bodies. Liver parenchyma, blood vessels, skin, and nerves are all practical targets for mimicking [70,71,72,73].

The physiological functions of the natural human kidneys include numerous metabolic and endocrinological functions. Erythropoietin generation [20], vitamin D synthesis [38], ammoniogenesis [39,40], glutathione metabolism [57], immunoregulatory support, and cytokine homeostasis [43] are all crucial for human physiology and potentially associated with elevation of mortality and morbidity of patients under long-term hemodialysis. Many clinical trials are ongoing for the practice of bioartificial kidneys produced by renal tubule cells cultured on semipermeable hollow fiber membranes. Some of them showed promising results such as reduced septic shock [74,75], appropriate inflammatory cytokine response [43], acceptable safety [75,76], and improved vitamin D metabolism [77].

To achieve good cell adhesion, different extracellular matrices, including laminin, pronectin, gelatin, collagen IV, or L-3,4-dihydroxyphenylalanine, are coated on the outer surfaces of hollow-fibered membranes [78,79,80,81], nevertheless, the coated materials could block the dialyzer membrane pores and impair the hemodialysis efficiency. Various addictive materials in synthetic membranes have been used in an attempt to overcome this problem. Graphene oxide (GO) or d-α-Tocopheryl polyethylene glycol 1000 succinate (TPGS) doped hollow-fibered membranes showed excellent cell adhesion and dialysis performance [82,83,84,85].

In addition to extracorporeal hollow-fibered bioartificial kidneys, implanted cell bioreactors are also under development. Cell source is a major challenge for cell bioreactors. Autologous and non-autologous renal cells are both reasonable choices theoretically. Autologous renal cells eliminate the issue of rejection induced by host immunity. However, the individualized cell incubation process increases the costs and unpredictability of bioartificial kidney performance. Non-autologous renal cells allow more standardized production after stable cell line establishment. To protect the implanted renal cells from host immunity, a barrier to prevent host immunity is necessary. The barrier needs to be a semipermeable membrane, which is crossable for oxygenation, nutrients, cytokines, or substances metabolized by the implanted renal cells. Meanwhile, antibodies and immune cells from patients must be blocked out of the cell bioreactor. Cell encapsulation with ultrathin synthetic membranes to prevent entry of antibodies and immune cells is a possible solution, but the encapsulation also limited the long-term cell viability [86]. Short-term renal cell therapy with hollow-fibered bioreactors is used in some clinical projects [43,87]. Silicon nanopore membranes fabricated with microelectromechanical systems also provide good protection from host immunity [88]. Except cell protection and cell sources, the challenge of uniform and steady cell production, good cell adhesion, and nutritional support of cells are all focuses of future development.

### 5.3. Dialysate Regeneration

To achieve miniaturization of blood purification systems, the large volume of dialysate is the most important challenge. In a 4 h hemodialysis course, up to 120 L of dialysate is needed. Requirement of mechanical pumps for high dialysate flow, stable source of dialysate, and large amount of clean water limits the possibility of miniaturization of the blood purification system.

To reduce the dialysate volume requirement in hemodialysis, dialysate regeneration is a crucial part for the miniaturization of blood purification systems. The REcirculation of DialYsate (REDY) system was first introduced in 1970 [89,90,91] to regenerate dialysate and reduce the required volume of water during dialysis. The water-efficient and portable features made the REDY system a promising dialysis mode.

**Figure 2:**
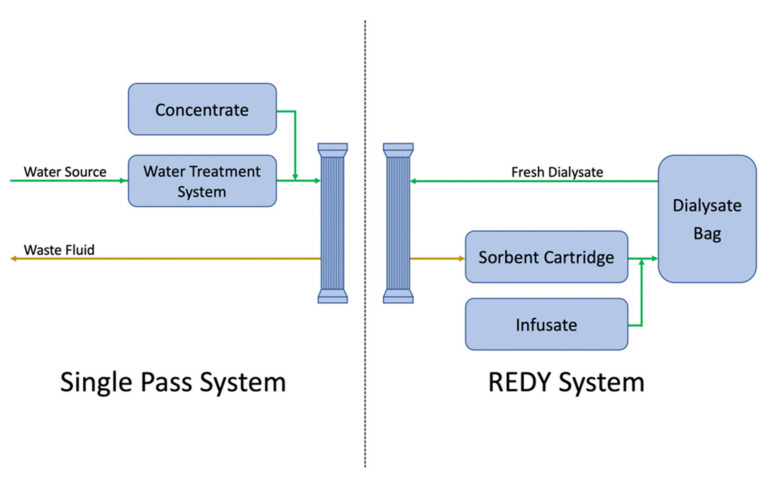
Comparison of the single pass system and the REDY system: dialysis with the single pass system requires a steady water source and used dialysate is abandoned after dialysis. In comparison, the REDY system regenerates fresh dialysate with sorbent cartridge.

During dialysis, dialysate and blood pass through artificial kidneys in a counter current pattern. When dialysate leaves the artificial kidney, multiple uremic toxins and excessive fluid from the blood compartment are withdrawn with dialysate. In a conventional single pass dialysis, the waste fluid is usually abandoned directly. Compared to single pass dialysis, the REDY system recirculates the waste fluid to an exchangeable sorbent cartridge to remove excessive solutes and regenerate dialysate (Figure 2). The sorbent cartridge contains materials such as charcoal, urease, or zirconium phosphate to eliminate the substance through chemical break down, ion exchange, or adsorptions [92]. The dialysate regeneration reduces the water requirement from 120 to 6 L per treatment [92,93].

The REDY system is not a new idea. Early versions of the REDY system were abandoned because of aluminum release, ammonia spill over, slow increase in sodium concentration of dialysate, and expensive cost of the system [92]. However, with the introduction of zirconium as ion exchangers and sorbent material, the adjusted size of sorbent cartridge, and modified dialysate components, these disadvantages could be overcome. In combination with the utility of nanotechnology, the dialysate requirement could be reduced to a greater extent.

Nanotechnology, tissue engineering, and dialysate regeneration encompassed the three key components for future artificial kidneys. With nanotechnology, artificial kidneys could be much smaller, more efficient, and driven by blood circulation without mechanical pumps. Combined with the REDY system, the fluid requirement for dialysis could be reduced significantly. Tissue engineering with renal tubule cells provides bioactivities which are essential to human bodies but can never be provided by conventional hemodialysis.

## 6. Next Generation Artificial Kidneys

### 6.1. Wearable Artificial Kidneys

After decades of development, the system of hemodialysis evolves gradually to a routine treatment. However, the limitation of a patient’s lifestyle and frequent visits to dialysis facilities still bother patients greatly. Thus, home dialysis was developed and improved patient quality of life significantly [94,95]. 

Compared to home dialysis, wearable artificial kidneys put dialysis treatment to another level with more convenience and less hemodynamic impacts. To be truly wearable, the artificial kidney must be light and small. In addition to miniaturization, the requirement of steady blood vessel access, better biocompatibility (including cytocompatibility, immunocompatibility, and hemocompatibility), and patient education are all barriers for the development of wearable artificial kidneys. When being wearable, the dialysis device needs to be operated by the patients themselves. During the dialysis treatment, a patient’s activity might lead to disconnection of the device from blood vessels and monitoring for blood leakage is required for safety concerns.

Because of prolonged dialysis, the importance of biocompatibility also increases. Blood clotting and complement pathway activation caused by membranes would increase complications after prolonged dialysis. Membrane engineering for excellent biocompatibility is crucial. The Center for Dialysis Innovation (CDI) at the University of Washington, WA, USA, are developing two possible solutions for biocompatibility [96]. Fluoropolymer, a material used in blood content devices, can bind fibrinogen in an inactive form and albumin tightly to avoid further clotting and immune reaction. Another solution is to create surfaces with super non-fouling properties that repel proteins so that protein fouling and coagulation do not occur [97]. Modification of the membrane surface with microelectromechanical systems to prevent protein fouling is also ongoing [59].

**Figure 3:**
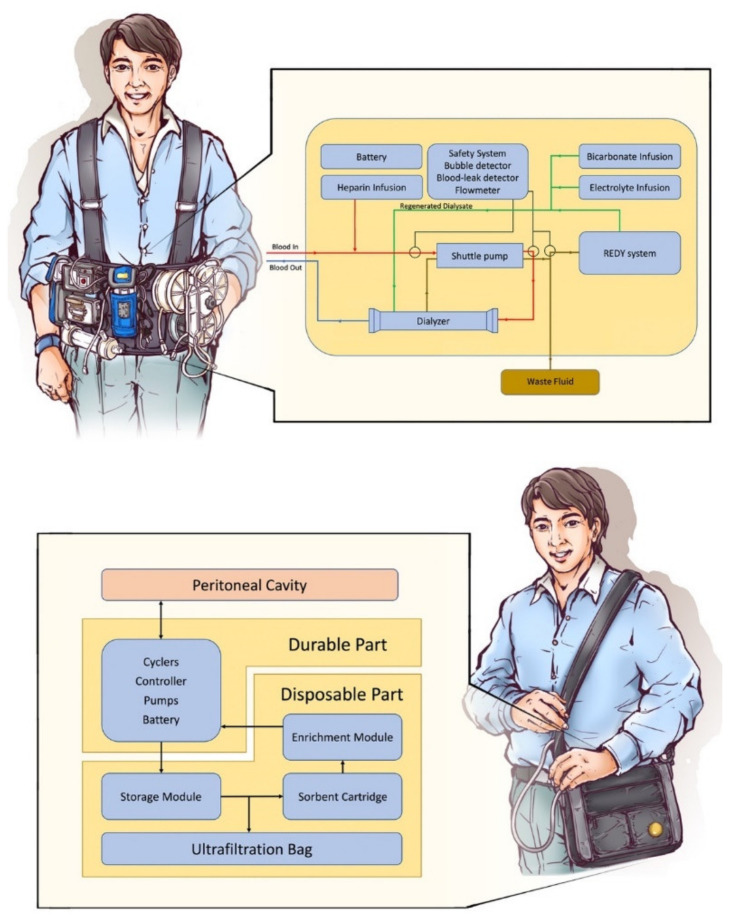
Basic concepts of a wearable hemodialyzer and a peritoneal-based wearable artificial kidney: within a wearable hemodialyzer (upper), the pumps, battery, safety system, and REDY system are all installed in a vest. During dialysis, the REDY system regenerates dialysate and reduces the requirement of water. A bubble detector, blood leak detector, and flowmeter monitor all fluid compartments and shut off the system in the case of an emergent condition. The peritoneal-based wearable artificial kidney (lower) is connected to the patient’s peritoneal cavity and regenerates dialysate fluid. The disposable part of the device is exchanged after dialysis.

A wearable hemodialysis device is under development by Gura and his colleagues at the University of California, Los Angeles (UCLA) [46]. A hollow-fibered dialyzer with a surface area of 0.2 m^2^, dialysate regeneration system, heparin and electrolyte reservoirs, and 375 mL of dialysate are all contained in a wearable dialysis device (Figure 3) with a weight of less than 4.5 kg.

In 2016, Gura conducted an FDA-approved human trial of the wearable artificial kidney. A 24 h dialysis with the wearable device was performed successfully in five patients without adverse effects. Mean urea, creatinine, and phosphorus clearances over 24 h were 17 ± 10, 16 ± 8, and 15 ± 9 mL/min, respectively. Mean β2-microglobulin clearance was 5 ± 4 mL/min [98]. Although the trial was discontinued due to device-related technical problems, including excessive carbon dioxide bubbles in the dialysate circuit and variable blood and dialysate flows, the trial still proved the possibility of wearable artificial kidneys.

Another design of wearable artificial kidneys is a modification based on the treatment with peritoneal dialysis. Although peritoneal dialysis is associated with less life-style restriction for patients compared with hemodialysis, storage and transportation of dialysate are still an issue for patients and limit their daily activities. Several peritoneal dialysis-based wearable devices are under development currently including the AWAK project by AWAK Technologies (Singapore) [53], the WEAKID project by Nanodialysis Inc (Oirschot, The Netherlands) [52,99], and CarryLife project by Triomed (Lund, Sweden) [51].

Peritoneal dialysis-based wearable artificial kidneys are composed of portable a sorbent cartridge and a dialysate reservoir (Figure 3). When connected to the filled peritoneal cavity, wasted dialysate flows into the sorbent cartridge and regenerates fresh dialysate intermittently. Without complicated parts needed for hemodialysis, the peritoneal dialysis based wearable artificial kidney could be miniaturized to purse size. Several human trials for peritoneal dialysis-based wearable devices are still under study. Current published research revealed sufficient dialysis clearance without severe adverse events [53,99]. Abdominal discomfort is the most common side effect [53].

### 6.2. Implantable Artificial Kidney

Among all renal replacement therapies, kidney transplantation offers the best quality of life, total financial costs, and survival rate [100,101,102]. However, the supplies of donated kidneys are far less than the demands. Most patients with end stage renal disease therefore sustain their lives with hemodialysis or peritoneal dialysis.

The implantable artificial kidney is a promising and challenging idea that emerged with the development of renal replacement therapy. The artificial kidney needs to fit several properties to be implantable and replace the function of human kidneys.

First, the requirement of energy supply needs to be as low as possible. An experiment in pigs revealed that the high efficiency of the silicon nanopore membrane allows the dialysis to be performed without mechanical pumps for blood compartments [64]. Blood circulation is one of the most sustained power sources in human bodies. If the artificial kidney is still depending on a battery for energy supply, the battery should be able to function for years to avoid frequent surgery to exchange the batteries.

Second, hemofiltration should be the modality of renal replacement rather than hemodialysis. Without the demand of dialysate, the implantable artificial kidney could be designed without mechanical pumps for dialysate. The requirement of a steady supply of dialysate, implanted inlet for dialysate, and large amount of fresh water will not exist anymore, then the artificial kidney could be truly implantable.

Third, the device should be extremely biocompatible. The replacement therapy with implantable artificial kidneys performed continuously all day after implantation. Coagulation and complement activation would be more severe than conventional intermittent hemodialysis. Continuous infusion of heparin is also impractical. Modification of the membrane surface should be done to absolutely prevent such reaction. Fluoropolymer material or membranes with super-non-fouling properties are possible solutions [59,96,97].

Last but not least, metabolic functions of natural renal cells have long been ignored in renal replacement therapy. The lack of metabolic function leads to multiple comorbidities in patients with end stage renal disease. Bioreactors with renal cells protected by nano-structured materials have been built for implantable artificial kidneys [103]. Moreover, the excellent protection from host immunity eliminates the requirement for immunosuppressants.

**Figure 4:**
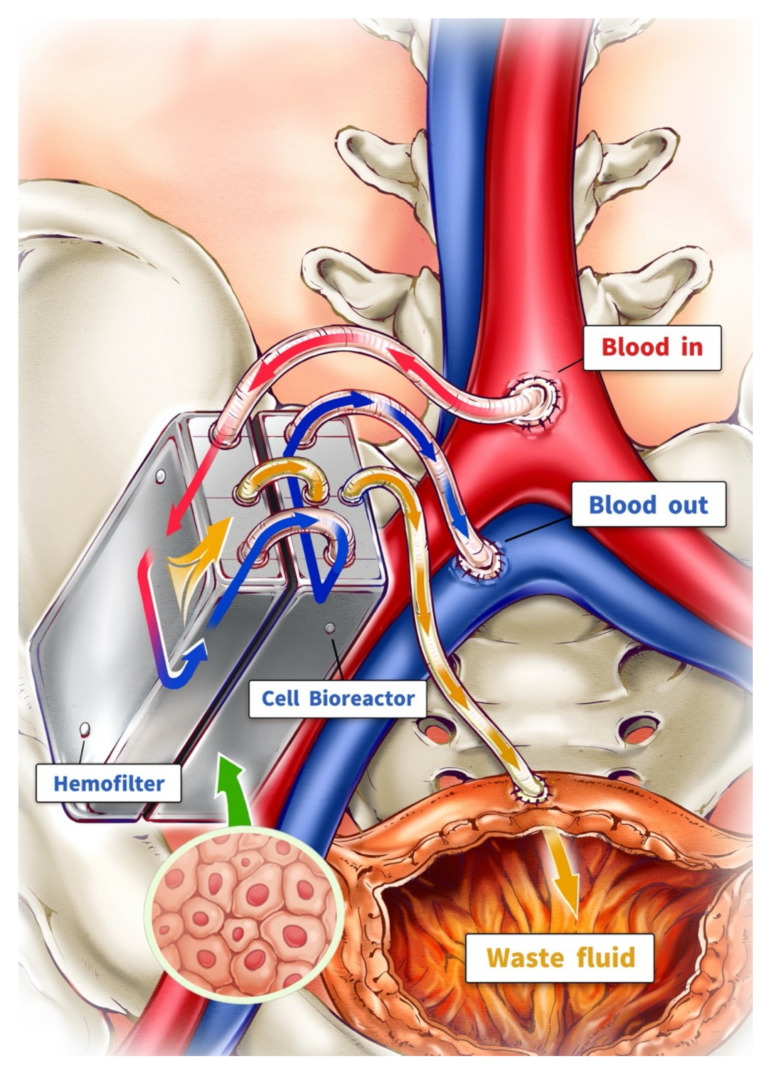
Implantable kidney: driven by the pressure difference in the artery and vein, blood passes through the implantable kidney, which is composed of a hemofilter and cell bioreactor. With a nanopore silicon membrane, waste fluid (yellow) created by hemofiltration flows into the patient’s urinary bladder. Dialyzed blood is transferred to the cell bioreactor after hemofiltration. Natural kidney metabolic function is restored by protected renal tubule cells in the cell bioreactor.

With these four properties, a truly implantable artificial kidney could be manufactured. The Kidney Project led by Shuvo Roy at the University of California San Francisco (UCSF) is currently developing a fist-sized implantable artificial kidney [50]. The artificial kidney consists of a bioreactor and a hemofilter connected to blood circulation with the common iliac vein and artery (Figure 4). The waste fluid is drained to the patient’s bladder as urination. The driving force of hemofiltration is based on the pressure difference between arterial and venous systems. After implantation, patients could theoretically live without any further hemodialysis.

## 7. Conclusions

The design of artificial kidneys has developed dramatically over decades after invention in the early 20th century. Hollow-fibered dialyzers overwhelmingly replace other artificial kidneys currently for the advantages of less fluid resistance and better surface area. However, patient’s quality of life and complications of artificial kidneys remain challenges of modern dialyzers. However, the time-consuming treatment course limits the patient’s quality of life significantly. Hemodialysis-related complications also highlight the unmet need for better biocompatible dialyzers. Multiple attempts, such as geometric designs and modified synthetic materials, have been made to overcome these challenges; nevertheless, there is still plenty of room for improvement. Miniaturization and restoration of kidney metabolic functions are the goals of new artificial kidneys. With the introduction of nanotechnology, tissue engineering, and the REDY system, the designs of modern artificial kidneys have changed dramatically. Although many technical problems still exist, wearable and implantable artificial kidneys are the next promising milestones in blood purification therapy.

## Data Availability

Not applicable.

## References

[1] Über H.G. (1928). On Blood Washing. Klin Wochenschr..

[2] Markwardt F. (2002). Hirudin as Alternative Anticoagulant—A Historical Review. Semin. Thromb. Hemost..

[3] Kolff W.J. (1965). First Clinical Experience with the Artificial Kidney. Ann. Intern. Med..

[4] Kurkus J., Ostrowski J. (2019). Nils Alwall and His Artificial Kidneys: Seventieth Anniversary of the Start of Serial Production. Artif. Organs.

[5] E Macneill A., E Doyle J., Anthone R., Anthone S. (1959). Technic with Parallel Flow, Straight Tube Blood Dialyzer. N. Y. State J. Med..

[6] (1927). The Isolation of Heparin from the Liver. Science.

[7] Brescia M.J., Cimino J.E., Appel K., Hurwich B.J. (1966). Chronic Hemodialysis Using Venipuncture and a Surgically Created Arteriovenous Fistula. N. Engl. J. Med..

[8] Ronco C. (2007). Fluid Mechanics and Crossfiltration in Hollow-Fiber Hemodialyzers. Contrib. Nephrol..

[9] Nakagawa S., Koshikawa S., Joh Y., Yamazaki M. (1977). Influence of dialysate flow pattern on solute clearance of hollow fiber kidney: A successful result with counter cross flow configuration. Trans Am Soc Artif Intern Organs..

[10] Von Hartitzsch B., Hoenich N.A., Samson P., Erickson J., Ashcroft R.A., Kerr D.N. (1973). A Clinical Evaluation of the New Dialysers. Kidney Int..

[11] Henne W., Dietrich W., Pelger M., Sengbusch G. (1984). Residual Ethylene Oxide in Hollow-Fiber Dialyzers. Artif. Organs.

[12] Henrich W.L. (2012). Principles and Practice of Dialysis.

[13] Ronco C., Brendolan A., Crepaldi C., Rodighiero M., Scabardi M. (2002). Blood and Dialysate Flow Distributions in Hollow-Fiber Hemodialyzers Analyzed by Computerized Helical Scanning Technique. J. Am. Soc. Nephrol..

[14] Hootkins R., Bourgeois B. (1991). The Effect of Ultrafiltration on Dialysance. Mathematical Theory and Experimental Verification. ASAIO Trans..

[15] Chanard J., Lavaud S., Randoux C., Rieu P. (2003). New Insights in Dialysis Membrane Biocompatibility: Relevance of Adsorption Properties and Heparin Binding. Nephrol. Dial. Transplant..

[16] Poppelaars F., Faria B., Da Costa M.G., Franssen C.F.M., Van Son W.J., Berger S.P., Daha M.R., Seelen M.A. (2018). The Complement System in Dialysis: A Forgotten Story?. Front. Immunol..

[17] Strohbach A., Busch R. (2021). Predicting the In Vivo Performance of Cardiovascular Biomaterials: Current Approaches In Vitro Evaluation of Blood-Biomaterial Interactions. Int. J. Mol. Sci..

[18] Clark W.R., Hamburger R.J., Lysaght M.J. (1999). Effect of Membrane Composition and Structure on Solute Removal and Biocompatibility in Hemodialysis. Kidney Int..

[19] Koga Y., Fujieda H., Meguro H., Ueno Y., Aoki T., Miwa K., Kainoh M. (2018). Biocompatibility of Polysulfone Hemodialysis Membranes and Its Mechanisms: Involvement of Fibrinogen and Its Integrin Receptors in Activation of Platelets and Neutrophils. Artif. Organs.

[20] Badve S.V., Beller E., Cass A., Francis D., Hawley C., MacDougall I.C., Perkovic V., Johnson D.W. (2013). Interventions for Erythropoietin-Resistant Anaemia in Dialysis Patients. Cochrane Database Syst. Rev..

[21] Hara H., Nakamura Y., Hatano M., Iwashita T., Shimizu T., Ogawa T., Kanozawa K., Hasegawa H. (2018). Protein Energy Wasting and Sarcopenia in Dialysis Patients. Contrib. Nephrol..

[22] Amadori A., Candi P., Sasdelli M., Massai G., Favilla S., Passaleva A., Ricci M. (1983). Hemodialysis Leukopenia and Complement Function with Different Dialyzers. Kidney Int..

[23] Liakopoulos V., Roumeliotis S., Gorny X., Dounousi E., Mertens P.R. (2017). Oxidative Stress in Hemodialysis Patients: A Review of the Literature. Oxidative Med. Cell. Longev..

[24] Said N., Lau W.J., Ho Y.-C., Lim S.K., Abidin M.N.Z., Ismail A.F. (2021). A Review of Commercial Developments and Recent Laboratory Research of Dialyzers and Membranes for Hemodialysis Application. Membranes.

[25] Sakai K. (1994). Determination of Pore Size and Pore Size Distribution: 2. Dialysis Membranes. J. Membr. Sci..

[26] Hayama M., Kohori F., Sakai K. (2002). AFM Observation of Small Surface Pores of Hollow-Fiber Dialysis Membrane Using Highly Sharpened Probe. J. Membr. Sci..

[27] Fukuda M., Saomoto H., Mori T., Yoshimoto H., Kusumi R., Sakai K. (2020). Impact of Three-Dimensional Tortuous Pore Structure on Polyethersulfone Membrane Morphology and Mass Transfer Properties from a Manufacturing Perspective. J. Artif. Organs.

[28] Duy Nguyen B.T., Nguyen Thi H.Y., Nguyen Thi B.P., Kang D.K., Kim J.F. (2021). The Roles of Membrane Technology in Artificial Organs: Current Challenges and Perspectives. Membranes.

[29] Chuasuwan A., Pooripussarakul S., Thakkinstian A., Ingsathit A., Pattanaprateep O. (2020). Comparisons of Quality of Life between Patients Underwent Peritoneal Dialysis and Hemodialysis: A Systematic Review and Meta-Analysis. Heal. Qual. Life Outcomes.

[30] Huang Z., Gao D., Letteri J.J., Clark W.R. (2009). Blood-Membrane Interactions during Dialysis. Semin. Dial..

[31] Huang J., Yi B., Li A.-M., Zhang H. (2015). Effects of Vitamin E-Coated Dialysis Membranes on Anemia, Nutrition and Dyslipidemia Status in Hemodialysis Patients: A Meta-Analysis. Ren. Fail..

[32] Sosa M.A., Balk E.M., Lau J., Liangos O., Balakrishnan V.S., Madias N.E., Pereira B.J.G., Jaber B.L. (2006). A Systematic Review of the Effect of the Excebrane Dialyser on Biomarkers of Lipid Peroxidation. Nephrol. Dial. Transplant..

[33] Yang S.-K., Xiao L., Xu B., Xu X.-X., Liu F.-Y., Sun L. (2014). Effects of Vitamin E-Coated Dialyzer on Oxidative Stress and Inflammation Status in Hemodialysis Patients: A Systematic Review and Meta-Analysis. Ren. Fail..

[34] Islam M.S., Hassan Z.A., Chalmin F., Vido S., Berrada M., Verhelst D., Donnadieu P., Moranne O., Esnault V.L. (2016). Vitamin E–Coated and Heparin-Coated Dialyzer Membranes for Heparin-Free Hemodialysis: A Multicenter, Randomized, Crossover Trial. Am. J. Kidney Dis..

[35] D’Arrigo G., Baggetta R., Tripepi G., Galli F., Bolignano D. (2016). Effects of Vitamin E-Coated versus Conventional Membranes in Chronic Hemodialysis Patients: A Systematic Review and Meta-Analysis. Blood Purif..

[36] Rocco M.V., Lockridge R.S., Beck G.J., Eggers P.W., Gassman J.J., Greene T., Larive B., Chan C.T., Chertow G.M., Copland M. (2011). The Effects of Frequent Nocturnal Home Hemodialysis: The Frequent Hemodialysis Network Nocturnal Trial. Kidney Int..

[37] Culleton B.F., Walsh M., Klarenbach S.W., Mortis G., Scott-Douglas N., Quinn R.R., Tonelli M., Donnelly S., Friedrich M.G. (2007). Effect of Frequent Nocturnal Hemodialysis vs. Conventional Hemodialysis on Left Ventricular Mass and Quality of Life: A Randomized Controlled Trial. JAMA.

[38] Jean G., Souberbielle J.C., Chazot C. (2017). Vitamin D in Chronic Kidney Disease and Dialysis Patients. Nutrition.

[39] Wesson D.E., Buysse J.M., Bushinsky D.A. (2020). Mechanisms of Metabolic Acidosis–Induced Kidney Injury in Chronic Kidney Disease. J. Am. Soc. Nephrol..

[40] Weiner I.D., Verlander J.W. (2013). Renal Ammonia Metabolism and Transport. Compr. Physiol..

[41] Abbott W., Bridges R.J., Meister A. (1984). Extracellular Metabolism of Glutathione Accounts for Its Disappearance from the Basolateral Circulation of the Kidney. J. Biol. Chem..

[42] Jelkmann W. (2011). Regulation of Erythropoietin Production. J. Physiol..

[43] Fissell W.H., Dyke D.B., Weitzel W.F., Buffington D.A., Westover A., MacKay S.M., Gutierrez J.M., Humes H.D. (2002). Bioartificial Kidney Alters Cytokine Response and Hemodynamics in Endotoxin-Challenged Uremic Animals. Blood Purif..

[44] Davenport A., Gura V., Ronco C., Beizai M., Ezon C., Rambod E. (2007). A Wearable Haemodialysis Device for Patients with End-Stage Renal Failure: A Pilot Study. Lancet.

[45] Shaldon S., Lysaght M. (2008). A Wearable Hemofilter for Continuous Ambulatory Ultrafiltration. Kidney Int..

[46] Gura V., Beizai M., Ezon C., Polaschegg H.-D. (2005). Continuous Renal Replacement Therapy for End-Stage Renal Disease. Contrib. Nephrol..

[47] Gura V., Macy A.S., Beizai M., Ezon C., Golper T.A. (2009). Technical Breakthroughs in the Wearable Artificial Kidney (WAK). Clin. J. Am. Soc. Nephrol..

[48] Gura V., Davenport A., Beizai M., Ezon C., Ronco C. (2009). Beta2-Microglobulin and Phosphate Clearances Using a Wearable Artificial Kidney: A Pilot Study. Am. J. Kidney Dis..

[49] Lee D.B.N., Roberts M. (2008). A Peritoneal-Based Automated Wearable Artificial Kidney. Clin. Exp. Nephrol..

[50] Shuvo Roy UoCSFU (2021). The Kidney Project. https://pharm.ucsf.edu/kidney/.

[51] Triomed (2021). CLR. Steady Concentration Peritoneal Dialysis—SCPD.

[52] Gerritsen K. (2018). WEAKID—Clinical Validation of Miniature Wearable Dialysis Machine—H2020. Impact.

[53] Htay H., Gow S.K., Jayaballa M., Oei E.L., Chan C., Wu S., Foo M.W. (2021). Preliminary Safety Study of the Automated Wearable Artificial Kidney (AWAK) in Peritoneal Dialysis Patients. Perit. Dial. Int..

[54] Group F.T. (2010). In-Center Hemodialysis Six Times per Week versus Three Times per Week. N. Engl. J. Med..

[55] Jardine M.J., Zuo L., Gray N., De Zoysa J.R., Chan C.T., Gallagher M.P., Monaghan H., Grieve S.M., Puranik R., Snelling P. (2017). A Trial of Extending Hemodialysis Hours and Quality of Life. J. Am. Soc. Nephrol..

[56] Mathew A., McLeggon J.-A., Mehta N., Leung S., Barta V., McGinn T., Nesrallah G. (2018). Mortality and Hospitalizations in Intensive Dialysis: A Systematic Review and Meta-Analysis. Can. J. Kidney Heal. Dis..

[57] Santangelo F., Witko-Sarsat V., Drüeke T., Descamps-Latscha B. (2004). Restoring Glutathione as a Therapeutic Strategy in Chronic Kidney Disease. Nephrol. Dial. Transplant..

[58] Kim S., Roy S. (2013). Microelectromechanical Systems and Nephrology: The Next Frontier in Renal Replacement Technology. Adv. Chronic Kidney Dis..

[59] Li L., Marchant R.E., Dubnisheva A., Roy S., Fissell W.H. (2011). Anti-Biofouling Sulfobetaine Polymer Thin Films on Silicon and Silicon Nanopore Membranes. J. Biomater. Sci. Polym. Ed..

[60] Zhu J., Marchant R.E. (2006). Dendritic Saccharide Surfactant Polymers as Antifouling Interface Materials to Reduce Platelet Adhesion. Biomacromolecules.

[61] Papra A., Gadegaard N., Larsen N.B. (2001). Characterization of Ultrathin Poly(Ethylene Glycol) Monolayers on Silicon Substrates. Langmuir.

[62] Popat K.C., A Desai T. (2004). Poly(Ethylene Glycol) Interfaces: An Approach For enhanced Performance of Microfluidic Systems. Biosens. Bioelectron..

[63] Muthusubramaniam L., Lowe R., Fissell W.H., Li L., Marchant R.E., Desai T.A., Roy S. (2011). Hemocompatibility of Silicon-Based Substrates for Biomedical Implant Applications. Ann. Biomed. Eng..

[64] Kim S., Feinberg B., Kant R., Chui B., Goldman K., Park J., Moses W., Blaha C., Iqbal Z., Chow C. (2016). Diffusive Silicon Nanopore Membranes for Hemodialysis Applications. PLoS ONE.

[65] Iqbal Z., Kim S., Moyer J., Moses W., Abada E., Wright N., Kim E.J., Park J., Fissell W.H., Vartanian S. (2019). In vitro and in vivo hemocompatibility assessment of ultrathin sulfobetaine polymer coatings for silicon-based implants. J. Biomater. Appl..

[66] Fissell W.H., Dubnisheva A., Eldridge A.N., Fleischman A.J., Zydney A.L., Roy S. (2009). High-Performance Silicon Nanopore Hemofiltration Membranes. J. Membr. Sci..

[67] Conlisk A.T., Datta S., Fissell W.H., Roy S. (2009). Biomolecular Transport through Hemofiltration Membranes. Ann. Biomed. Eng..

[68] Kanani D.M., Fissell W.H., Roy S., Dubnisheva A., Fleischman A., Zydney A.L. (2010). Permeability–Selectivity Analysis for Ultrafiltration: Effect of Pore Geometry. J. Membr. Sci..

[69] Vacanti J.P., Morse M.A., Saltzman W.M., Domb A.J., Perez-Atayde A., Langer R. (1988). Selective Cell Transplantation Using Bioabsorbable Artificial Polymers as Matrices. J. Pediatr. Surg..

[70] Hosseini V., Maroufi N.F., Saghati S., Asadi N., Darabi M., Ahmad S.N.S., Hosseinkhani H., Rahbarghazi R. (2019). Current Progress in Hepatic Tissue Regeneration by Tissue Engineering. J. Transl. Med..

[71] Vig K., Chaudhari A., Tripathi S., Dixit S., Sahu R., Pillai S., Dennis V.A., Singh S.R. (2017). Advances in Skin Regeneration Using Tissue Engineering. Int. J. Mol. Sci..

[72] Gu X., Ding F., Williams D.F. (2014). Neural Tissue Engineering Options for Peripheral Nerve Regeneration. Biomaterials.

[73] Pearson R.G., Bhandari R., Quirk R.A., Shakesheff K.M. (2017). Recent Advances in Tissue Engineering. J. Long-Term Eff. Med. Implant..

[74] Humes H.D., Buffington D.A., Lou L., Abrishami S., Wang M., Xia J., Fissell W.H. (2003). Cell Therapy with a Tissue-Engineered Kidney Reduces the Multiple-Organ Consequences of Septic Shock. Crit. Care Med..

[75] Ding F., Yevzlin A., Humes H. (2010). A Selective Cytopheretic Inhibitory Device (SCD) Accelerates Renal Recovery and Improves Mortality in ICU Patients with AKI and MOF in an Exploratory Clinical Study. ASAIO Ren. Abstr..

[76] Humes H.D., Weitzel W.F., Bartlett R.H., Swaniker F.C., Paganini E.P., Luderer J.R., Sobota J. (2004). Initial Clinical Results of the Bioartificial Kidney Containing Human Cells in ICU Patients with Acute Renal Failure. Kidney Int..

[77] Humes H.D., Mackay S.M., Funke A.J., Buffington D.A. (1999). Tissue Engineering of a Bioartificial Renal Tubule Assist Device: In Vitro Transport and Metabolic Characteristics. Kidney Int..

[78] Schophuizen C.M., De Napoli I.E., Jansen J., Teixeira S., Wilmer M.J., Hoenderop J.G., Heuvel L.P.V.D., Masereeuw R., Stamatialis D. (2015). Development of a Living Membrane Comprising a Functional Human Renal Proximal Tubule Cell Monolayer on Polyethersulfone Polymeric Membrane. Acta Biomater..

[79] Ni M., Teo J., Ibrahim M.S., Zhang K., Tasnim F., Chow P.-Y., Zink D., Ying J. (2011). Characterization of Membrane Materials and Membrane Coatings for Bioreactor Units of Bioartificial Kidneys. Biomaterials.

[80] Oo Z.Y., Deng R., Hu M., Ni M., Kandasamy K., Ibrahim M.S., Ying J.Y., Zink D. (2011). The Performance of Primary Human Renal Cells in Hollow Fiber Bioreactors for Bioartificial Kidneys. Biomaterials.

[81] Zhang H., Tasnim F., Ying J.Y., Zink D. (2009). The Impact of Extracellular Matrix Coatings on the Performance of Human Renal Cells Applied in Bioartificial Kidneys. Biomaterials.

[82] Modi A., Verma S.K., Bellare J. (2018). Graphene Oxide-Doping Improves the Biocompatibility and Separation Performance of Polyethersulfone Hollow Fiber Membranes for Bioartificial Kidney Application. J. Colloid Interface Sci..

[83] Modi A., Verma S.K., Bellare J. (2017). Graphene Oxide Nanosheets and D-α-Tocopheryl Polyethylene Glycol 1000 Succinate (TPGS) Doping Improves Biocompatibility and Ultrafiltration in Polyethersulfone Hollow Fiber Membranes. J. Colloid Interface Sci..

[84] Modi A., Verma S.K., Bellare J. (2018). Extracellular Matrix-Coated Polyethersulfone-TPGS Hollow Fiber Membranes Showing Improved Biocompatibility and Uremic Toxins Removal for Bioartificial Kidney Application. Colloids Surf. B Biointerfaces.

[85] Modi A., Verma S.K., Bellare J.R. (2020). Surface-Functionalized Poly(Ether Sulfone) Composite Hollow Fiber Membranes with Improved Biocompatibility and Uremic Toxins Clearance for Bioartificial Kidney Application. ACS Appl. Bio. Mater..

[86] Orive G., Hernandez R.M., Gascón A.R., Calafiore R., Chang T.M., Vos P.D., Hortelano G., Hunkeler D., Lacik I., Shapiro A.J. (2003). Cell Encapsulation: Promise and Progress. Nat. Med..

[87] Humes H.D., Buffington D.A., MacKay S.M., Funke A.J., Weitzel W.F. (1999). Replacement of Renal Function in Uremic Animals with a Tissue-Engineered Kidney. Nat. Biotechnol..

[88] Roy S., Dubnisheva A., Eldridge A., Fleischman A.J., Goldman K.G., Humes H.D., Zydney A.L., Fissell W.H. Silicon Nanopore Membrane Technology for an Implantable Artificial Kidney. Proceedings of the TRANSDUCERS 2009—2009 International Solid-State Sensors, Actuators and Microsystems Conference.

[89] Gordon A., Better O.S., A Greenbaum M., Marantz L.B., Gral T., Maxwell M.H. (1971). Clinical Maintenance Hemodialysis with a Sorbent-Based, Low-Volume Dialysate Regeneration System. Trans.—Am. Soc. Artif. Intern. Organs.

[90] Blagg C.R., E Vizzo J., Jensen W.B., Cole J.J. (1974). Experience with a Sorbent-Based Dialysate Regeneration System for Hemodialysis. Prog. Biochem. Pharmacol..

[91] Blumenkrantz M.J., Gordon A., Roberts M., Lewin A.J., Peeker E.A., Moran J.K., Coburn J.W., Maxwell M.H. (2008). Applications of the Redy^®^ Sorbent System to Hemodialysis and Peritoneal Dialysis. Artif. Organs.

[92] Agar J.W. (2010). Review: Understanding Sorbent Dialysis Systems. Nephrology.

[93] Ash S.R. (2009). Sorbents in Treatment of Uremia: A Short History and a Great Future. Semin. Dial..

[94] Jha C.M. (2021). Cost-Effectiveness of Home Hemodialysis with Bedside Portable Dialysis Machine “DIMI” in the United Arab Emirates. Cureus.

[95] Kitsche B., Bach D. (2021). Home Hemodialysis. Nephrologe.

[96] Himmelfarb J., Ratner B. (2020). Wearable Artificial Kidney: Problems, Progress and Prospects. Nat. Rev. Nephrol..

[97] Zhang Z., Zhang M., Chen S., Horbett T.A., Ratner B.D., Jiang S. (2008). Blood Compatibility of Surfaces with Superlow Protein Adsorption. Biomaterials.

[98] Gura V., Rivara M.B., Bieber S., Munshi R., Smith N.C., Linke L., Kundzins J., Beizai M., Ezon C., Kessler L. (2016). A Wearable Artificial Kidney for Patients with End-Stage Renal Disease. JCI Insight..

[99] Van Gelder M.K., Ligabue G., Giovanella S., Bianchini E., Simonis F., Hazenbrink D.H., Joles J.A., Bajo Rubio M.A., Selgas R., Cappelli G. (2020). In Vitro Efficacy and Safety of a System for Sorbent-Assisted Peritoneal Dialysis. Am. J. Physiol.-Ren. Physiol..

[100] Johansen K.L., Chertow G.M., Foley R.N., Gilbertson D.T., Herzog C.A., Ishani A., Israni A.K., Ku E., Tamura M.K., Li S. (2021). US Renal Data System 2020 Annual Data Report: Epidemiology of Kidney Disease in the United States. Am. J. Kidney Dis..

[101] Finkelstein F.O., Schiller B., Daoui R., Gehr T.W., Kraus M.A., Lea J., Lee Y., Miller B.W., Sinsakul M., Jaber B.L. (2012). At-Home Short Daily Hemodialysis Improves the Long-Term Health-Related Quality of Life. Kidney Int..

[102] Kimmel P.L., Peterson R.A., Weihs K.L., Simmens S.J., Alleyne S., Cruz I., Veis J.H. (2000). Multiple Measurements of Depression Predict Mortality in a Longitudinal Study of Chronic Hemodialysis Outpatients. Kidney Int..

[103] Fissell W.H., Manley S., Westover A., Humes D., Fleischman A.J., Roy S. (2006). Differentiated Growth of Human Renal Tubule Cells on Thin-Film and Nanostructured Materials. ASAIO J..

